# 1α,25(OH)_2_D_3_ Suppresses the Migration of Ovarian Cancer SKOV-3 Cells through the Inhibition of Epithelial–Mesenchymal Transition

**DOI:** 10.3390/ijms17081285

**Published:** 2016-08-19

**Authors:** Yong-Feng Hou, Si-Hai Gao, Ping Wang, He-Mei Zhang, Li-Zhi Liu, Meng-Xuan Ye, Guang-Ming Zhou, Zeng-Li Zhang, Bing-Yan Li

**Affiliations:** 1 Department of Toxicology, School of Public Health, Soochow University, Suzhou 215123, China; 20144247024@stu.suda.edu.cn; 2 Department of Nutrition and Food Hygiene, Wenzhou Center for Disease Control and Prevention, Wenzhou 325000, China; gaosihai1987@163.com; 3 Organ Transplant Institute, Fuzhou General Hospital, Fuzhou 350025, China; pingwangsuda@163.com; 4 AIDS-STDs Prevention and Control Department, Wenzhou Center for Disease Control and Prevention, Wenzhou 325000, China; zhanghemei0306@126.com; 5 State Key Laboratory of Oncology in South China, Collaborative Innovation Center for Cancer Medicine, Sun Yat-sen University Cancer Center, Guangzhou 510060, China; zhililiu2@sina.com; 6 Department of Nutrition and Food Hygiene, School of Public Health, Soochow University, Suzhou 215123, China; 20154247021@stu.suda.edu.cn; 7 Department of Radiation Biology, School of Radiation Medication and Protection, Soochow University, Suzhou 215123, China; gmzhou@suda.edu.cn; 8 Department of Labor Hygiene and Environmental Health, School of Public Health, Soochow University, Suzhou 215123, China

**Keywords:** vitamin D, ovarian cancer, migration, EMT

## Abstract

Ovarian cancer is the most lethal gynecological malignancy due to its high metastatic ability. Epithelial-mesenchymal transition (EMT) is essential during both follicular rupture and epithelium regeneration. However, it may also accelerate the progression of ovarian carcinomas. Experimental studies have found that 1α,25-dihydroxyvitamin-D3 [1α,25(OH)_2_D_3_] can inhibit the proliferation of ovarian cancer cells. In this study, we investigated whether 1α,25(OH)_2_D_3_ could inhibit the migration of ovarian cancer cells via regulating EMT. We established a model of transient transforming growth factor-β1(TGF-β1)-induced EMT in human ovarian adenocarcinoma cell line SKOV-3 cells. Results showed that, compared with control, 1α,25(OH)_2_D_3_ not only inhibited the migration and the invasion of SKOV-3 cells, but also promoted the acquisition of an epithelial phenotype of SKOV-3 cells treated with TGF-β1. We discovered that 1α,25(OH)_2_D_3_ increased the expression of epithelial marker E-cadherin and decreased the level of mesenchymal marker, Vimentin, which was associated with the elevated expression of VDR. Moreover, 1α,25(OH)_2_D_3_ reduced the expression level of transcription factors of EMT, such as slug, snail, and β-catenin. These results indicate that 1α,25(OH)_2_D_3_ suppresses the migration and invasion of ovarian cancer cells by inhibiting EMT, implying that 1α,25(OH)_2_D_3_ might be a potential therapeutic agent for the treatment of ovarian cancer.

## 1. Introduction

Ovarian cancer has the highest fatality rate among women, primarily due to advanced stage at diagnosis, the lack of effective therapies for late-stage, and the replase after chemotherapy and surgery, which result in poor overall survival for the patients [1]. About 90% of all ovarian cancers are epithelial ovarian cancer, which originated from ovarian surface epithelium. Hence, it is crucial to throw light on relevant molecular mechanisms of epithelial ovarian cancer progression for seeking targeted therapy that can help improve survival.

Epithelial-mesenchymal transition (EMT) is a reversible cellular process by which epithelial cells depolarize, lose cell–cell contacts, and gain a spindle-mesenchymal morphology. It is characterized by loss of epithelial morphology and cytoskeletal reorganization, rendering cells more migratory and invasive [2]. This process is essential for embryonic development and wound healing. The ovarian surface epithelium can transform back and forth between epithelial and mesenchymal phenotypes in both follicular rupture and subsequent ovarian remodeling [3]. Moreover, tumor cells, through EMT, can enhance invasion and acquire properties of cancer stem-like cells, secondary tumor-initiating and chemoresistance [4,5,6]. Recent research suggests that EMT plays a critical role in the progression of ovarian carcinomas [7,8,9]. Therefore, it is essential to develop newer therapeutic methods with complete efficacy and low-toxicity toward metastatic cancer.

Over the past two decades, vitamin D has been inspected preclinically for its efficacy in chemopreventive and anticancer therapy [7,8]. Experimental studies suggest that 1α,25(OH)_2_D_3_, active metabolite of vitamin D, and its synthetic derivatives, protect against ovarian cancer, manifesting anti-proliferative and pro-apoptotic effects in ovarian cancer cell lines [6,7,8,9,10,11,12] and anti-tumorigenesis in animal models [13,14,15]. However, the mechanisms of vitamin D inhibiting ovarian cancer remain largely unknown. The transforming growth factor (TGF)-β signaling pathway is a key inducer of EMT. In this study, we investigated whether 1α,25(OH)_2_D_3_ suppresses migration and invasion of human ovarian adenocarcinoma cell line SKOV-3 cells by regulating EMT. We found that TGF-β-induced EMT in ovarian cancer cells could be inhibited by 1α,25(OH)_2_D_3_.

## 2. Results

### 2.1. 1α,25(OH)_2_D_3_ Inhibits the Migration of Human Ovarian Cancer SKOV-3 Cells

**Figure 1:**
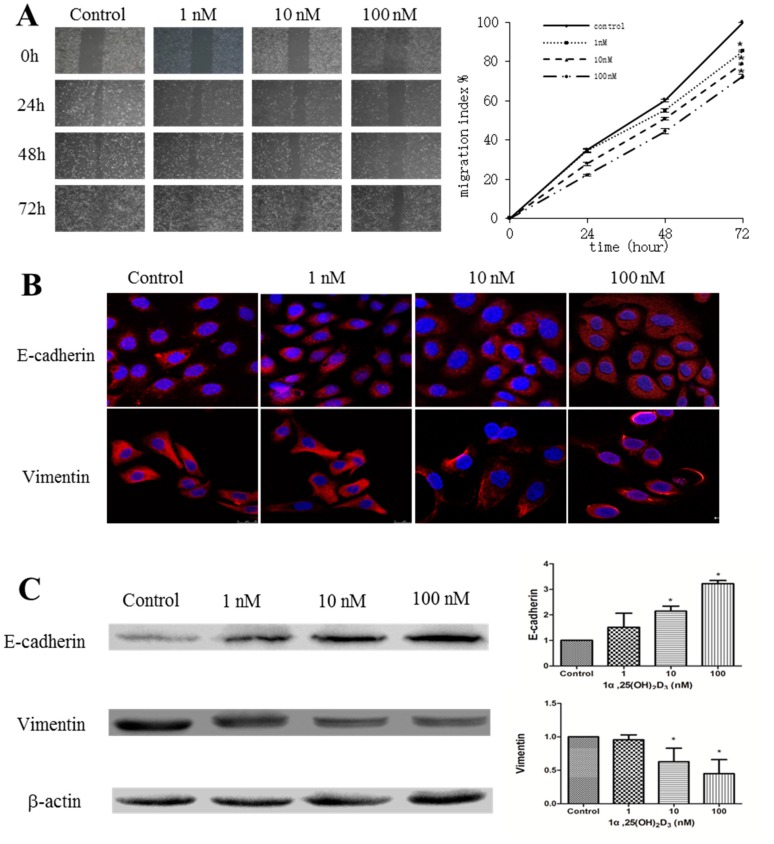
1α,25(OH)_2_D_3_ inhibited the migration of human ovarian adenocarcinoma cell line SKOV-3 cells. (**A**) **Left**: representative pictures of the wound area obtained 24, 48 and 72 h after scratching. 100× magnification; **Right**: migration index (%) = [(the initialized width of the scratch) − (the final width of the scratch)]/(the initialized width of the scratch); (**B**) representative pictures of E-cadherin and Vimentin were captured by confocal laser scanner microscopy (CLSM) 24 h after being treated with 1α,25(OH)_2_D_3_. Nuclear DNA was visualized by 4′,6-diamidino-2-phenylindole (DAPI) staining. 200× magnification; (**C**) **Left**: Western blot analysis of the indicated proteins in SKOV-3 cells. β-actin served as a loading control; **Right**: the level of the indicated protein was quantified with gray value. The data represent the Mean ± SD. * *p* < 0.05 versus control.

Our previous study has demonstrated that 1α,25(OH)_2_D_3_ inhibited the proliferation of SKOV-3 cells in a dose-dependent manner [12]. We wondered whether 1α,25(OH)_2_D_3_ could also inhibit the migration of these cells. After SKOV-3 cells were treated by 1, 10, or 100 nmol/L of 1α,25(OH)_2_D_3_, the cell migration decreased in both a time- and dose-dependent manner (Figure 1A, *p* < 0.05). Meanwhile, 1α,25(OH)_2_D_3_ shortened the moving distance and reduced the moving speed of SKOV-3 cells compared with control group (Supplementary Materials Figure S1). Furthermore, we analyzed the expression of E-cadherin and Vimentin, biomarkers of EMT. We found the increased expression of E-cadherin and decreased expression of Vimentin in the cytoplasm of SKOV-3 cells treated with 1α,25(OH)_2_D_3_ for 24 h (Figure 1C). The results of Western blotting also showed that the expression of E-cadherin was significantly increased, and Vimentin was clearly decreased when treated with 10 or 100 nmol/L of 1α,25(OH)_2_D_3_ (Figure 1C, *p* < 0.05). Collectively, 1α,25(OH)_2_D_3_ inhibited the migration of ovarian cancer cells, which was associated with the altered expression of E-cadherin and Vimentin.

### 2.2. Establishment of TGF-β1-Induced EMT in SKOV-3 Cells

**Figure 2:**
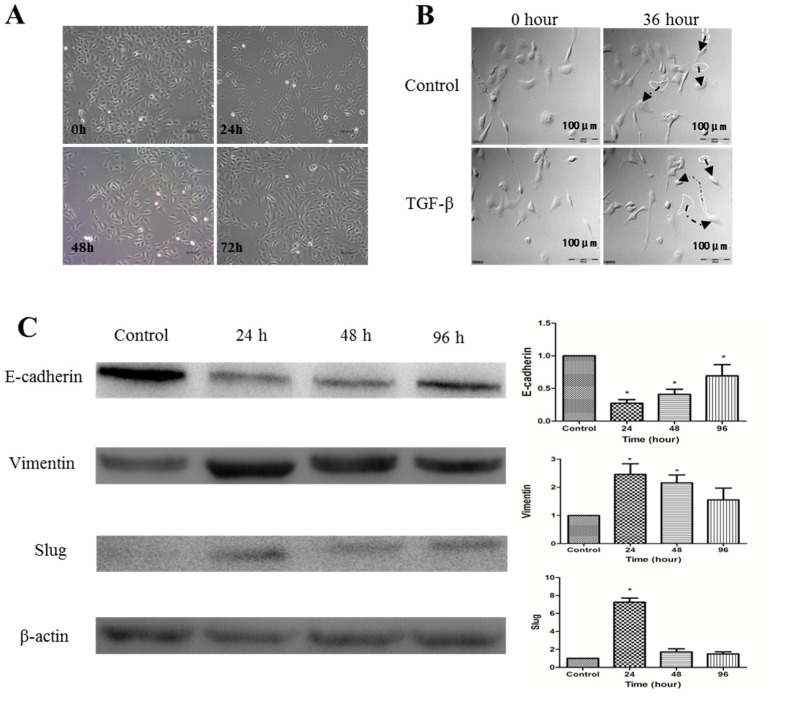
Transforming growth factor-β1 (TGF-β1) induces EMT of SKOV-3 cells. (**A**) SKOV-3 cells were exposed to 10 ng/mL of TGF-β1. Compared to the group of control, TGF-β1-treated SKOV-3 cells lost their cobblestone shape and adopted a fibroblast-like, spindle-shaped morphology. Morphology photographs were taken at 24, 48, and 72 h (magnification of 400×); (**B**) analyses with a Live Cell Imaging System showed that the movement distance increased further after SKOV-3 cells were treated with TGF-β1 for 36 h than control cells (Arrows refer to the movement track of SKOV-3 cells); (**C**) **Left**: Western blot analysis of the indicated proteins in SKOV-3 cells. β-actin served as a loading control; **Right**: the level of the indicated protein was quantified with gray value. The data represent the Mean ± SD. * *p* < 0.05 versus control.

Our findings that 1α,25(OH)_2_D_3_ increases the expression of E-cadherin and decreases that of Vimentin prompted us to study whether 1α,25(OH)_2_D_3_ inhibited EMT or not. Thus, we first established a model of TGF-β1-induced EMT of SKOV-3 cells. After being stimulated with 10 ng/mL TGF-β1 for 24 h, cell morphology changed from pebble-like epithelial to spindle-like mesenchymal, and it gradually elongated in 72 h (Figure 2A). The administration of TGF-β1 promoted cell migration and pseudopodium stretching frequency in 36 h (Figure 2B). Western blotting analyses confirmed the EMT phenotype of TGF-β1-treated SKOV-3 cells with decreased expression level of E-cadherin but increased Vimentin, compared with untreated cells (Figure 2C, *p* < 0.05). The expression of Slug, a transcription factor of EMT, rapidly increased in 24 h after cells were treated with TGF-β1 (*p* < 0.05). Taken together, results of morphological changes and protein expression patterns strongly indicated that we successfully established an experimental model of TGF-β1-induced EMT in ovarian cancer SKOV-3 cells.

### 2.3. 1α,25 (OH)_2_D_3_ Inhibited the Migration and Invasion of SKOV-3 Cells during TGF-β1-Induced EMT

**Figure 3:**
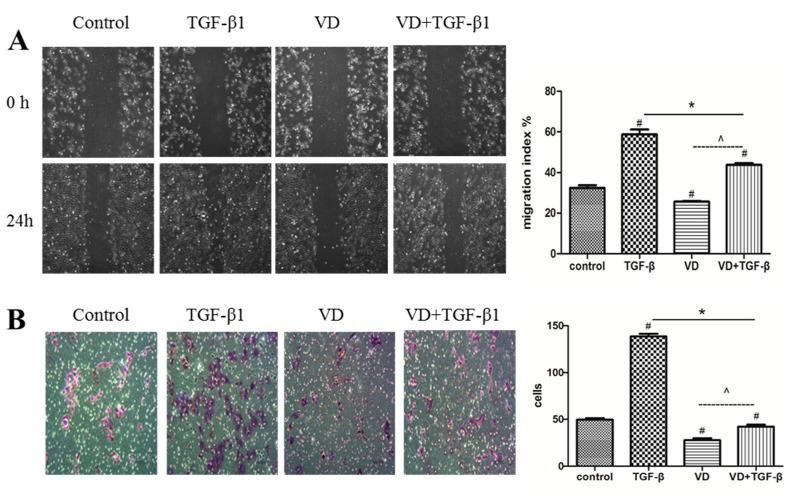
1α,25(OH)_2_D_3_ inhibited the migration and invasion of SKOV-3 cells during TGF-β1-induced EMT. (**A**) **Left**: representative pictures of the wound area obtained 24 h after scratching. 100× magnification; **Right**: migration index (%) = [(the initialized width of the scratch) − (the final width of the scratch)]/(the initialized width of the scratch); (**B**) **Left**: invasion assay was carried out using the 24-well Becton Dickinson (BD) Biocoat Matrigel Invasion Chambers(magnification of 400×); **Right**: the cells on the bottom of inserts were counted under microscope. # *p* < 0.05 versus negative control, * *p* < 0.05 versus TGF-β1, and ^ *p* < 0.05, 1α,25(OH)_2_D_3_ (VD) + TGF-β group versus VD group.

One of the functional changes of EMT is the increase in migration and invasion capacities, typically characteristics of mesenchymal cells. Thus, we determined whether 1α,25(OH)_2_D_3_ decreases cell migration and invasion accompanied with the TGF-β1-induced EMT. Compared with negative control, 1α,25(OH)_2_D_3_ alone significantly decreased the migration of SKOV-3 cells, while TGF-β dramatically increased the cell migration. However, the elevation of migration by TGF-β1 was significantly decreased by the treatment together with 1α,25(OH)_2_D_3_ (Figure 3A, *p* < 0.05). 1α,25(OH)_2_D_3_ similarly reversed the shortened motion tracking during TGF-β1-induced EMT, compared to 1α,25(OH)_2_D_3_-untreated cells (Supplementary Materials, Figure S2). Subsequently, we determined the invasion ability of cells in vitro after being treated with 1α,25(OH)_2_D_3_. As showed in Figure 3B, TGF-β1 dramatically increased the invasion ability of SKOV-3 cells, which were the most important characteristics of a metastatic cell. In contrast, 1α,25(OH)_2_D_3_ substantially inhibited the invasion. These results were consistent with data obtained by the cell migration assay, indicating that 1α,25(OH)_2_D_3_ had a greater inhibitory ability for migration and invasion of ovarian cancer cells.

### 2.4. 1α,25(OH)_2_D_3_ Regulates the Expression of EMT Markers in TGF-β1-Treated SKOV-3 Cells

**Figure 4:**
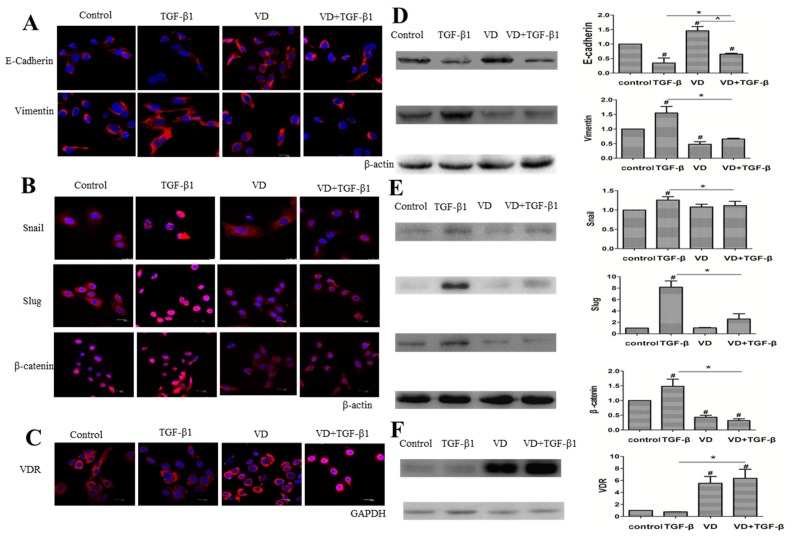
1α,25(OH)_2_D_3_ regulated the expression of EMT-related markers in SKOV-3 cells exposed to TGF-β1. (**A**–**C**) representative pictures of indicated proteins E-cadherin, Vimentin, Snail, Slug, β-catenin and VDR were captured by confocal laser scanner microscopy (CLSM). Nuclear DNA was visualized by DAPI staining. 200× magnification; (**D**–**F**) **Left**: Western blot analysis of the indicated proteins in SKOV-3 cells. β-actin or GAPDH served as a loading control; **Right**: the level of the indicated protein was quantified with gray value. The data represent the Mean ± SD. # *p* < 0.05 versus negative control, * *p* < 0.05 versus TGF-β1-treated group, and ^ *p* < 0.05, VD + TGF-β group versus VD group.

Subsequently, we wanted to determine whether 1α,25(OH)_2_D_3_ regulates the protein level of key EMT markers in TGF-β1-treated cells. Figure 4A showed representative images of immunofluorescence staining for E-cadherin and Vimentin in SKOV-3 cells. Compared to TGF-β1-treated cells, E-cadherin, the marker of epithelial cells, increased, while Vimentin, the marker of mesenchymal cells, decreased. Quantification of Western blotting results revealed that these alterations were significant at 1α,25(OH)_2_D_3_-treated cells when compared with the corresponding controls (Figure 4D, *p* < 0.05). These results indicated that the promotion of TGF-β1- induced EMT could be inhibited by 1α,25(OH)_2_D_3_. One of the EMT characteristics is the increased expression of EMT-related transcription factors including Snail, Slug and β-catenin. Figure 4B showed that the expression of snail, slug, and β-catenin increased in TGF-β1-treated cells. However, the administration of 1α,25(OH)_2_D_3_ resulted in the decrease of these compared to TGF-β1-treated cells. Western blotting results also showed a similar pattern of EMT-related proteins (Figure 4E, *p* < 0.05). These results demonstrated that 1α,25(OH)_2_D_3_ could reverse TGF-β1-induced EMT in SKOV-3 cells.

1α,25(OH)_2_D_3_ mediates target genes by binding to the vitamin D receptor (VDR). The results from both immunofluorescence and Western blotting showed that the expression of VDR in SKOV-3 cells only treated by TGF-β1 was lower than that in cells treated with the combination of 1α,25(OH)_2_D_3_ and TGF-β1 (Figure 4C,F, *p* < 0.05). These results showed that 1α,25(OH)_2_D_3_ inhibited the TGF-β1-induced EMT accompanied with increased expression of VDR in ovarian cancer cells.

## 3. Discussion

Ovarian cancer is the most lethal gynaecological cancer, and Epithelial-mesenchymal transition (EMT) was reported to be association with ovarian cancer cell dissemination and invasion [16]. To the best of our knowledge, this study is the first to report that 1α,25(OH)_2_D_3_ decreased the migration and invasion of human ovarian adenocarcinoma cell line SKOV-3 cells through inhibiting TGF-β1-induced EMT. This result is also in accordance with previous reports indicating the anti-metastasis potential of 1α,25(OH)_2_D_3_ in other types of cancer cells, including colon [15], breast [17], pancreatic [18], and lung cancers [19].

The normal ovarian surface epithelium exhibit epithelial and mesenchymal characteristics by the expression of both keratin and vimentin [20]. It is believed that ovarian surface epithelial cells adapt to changes by transitions between epithelial and mesenchymal stages during both follicular rupture and epithelium regeneration. Moreover, this plasticity may lie on the origin of ovarian cancer, an important initiating event in promoting tumor cell dissemination leading to metastasis [21]. We found that expression of both E-cadherin and Vimentin were observed in human ovarian cancer SKOV-3 cells. 1α,25(OH)_2_D_3_ inhibited migration of SKOV-3 cells accompanied with the decreased expression of E-cadherin but increased Vimentin. Furthmore, TGF-β1-induced EMT in ovarian cancer cells was also inhibited by 1α,25(OH)_2_D_3_.

TGF-β is a multifunctional cytokine that acts as a tumor suppressor in early stages through stopping proliferation, inducing differentiation, or promoting apoptosis but promotes tumor progression in late stages through multiple mechanisms, including inducing EMT in cancer cells [22]. In addition, TGF-β is frequently used as a key inducer of EMT for experimental models. Other than changes of epithelial and mesenchymal markers, EMT is characterized by altered location of transcription factors, such as β-catenin, Slug, Snail, Twist and Sox10. In the present study, TGF-β1 promoted the expression of Slug, Snail and β-catenin but also increased their localization in the nuclei of ovarian cancer cells. For another study, TGF-β1-induced EMT promotes breast cancer cell migration toward lymphatic endothelial cells by activating CCR7 [23]. Snail1 is a cofactor for Smad3/4 during TGF-β-induced EMT, and a strong correlation was also found between loss of CAR and E-cadherin and nuclear co-expression of Snail1 and Smad3/4 in breast cancer [24]. TGF-β-induced CD59 expression during EMT is dependent on Smad3 but not on Smad2 in lung cancer A549 cells [25]. Liu et al reported that the JAK/STAT3 pathway is required for TGF-β-induced EMT, and the IL-6/JAK/STAT3 and TGF-β/Smad signaling synergistically gain EMT in lung cancer [26]. Therefore, TGF-β-induced EMT as a model promotes metastasis of tumor cells through activating many pathways. We also included an established model of transient TGF-β1-induced EMT in human ovarian cancer cells. Furthermore, TGF-β1-induced EMT in ovarian cancer cells was inhibited by treatment with 1α,25(OH)_2_D_3_.

During recent years, vitamin D has been increasingly concerned as a potential for anti-cancer therapy, especially for the role of vitamin D in reducing risk and progression of colon, breast and prostate cancer [8,27,28]. However, there are fewer studies on the effect of vitamin D on proliferation and invasion of ovarian cancer. It is reported that Solar UVB irradiance, which resulted in higher level of 25-dihydroxyvitamin D [25(OH)D] in serum, a widely accepted biomarker of vitamin D status, was inversely associated with incidence rates of ovarian cancer in 175 countries in 2002. The high concentrations of the vitamin D receptor were demonstrated in ovarian cancer cells [27], and 1α,25(OH)_2_D_3_ has been shown to inhibit cell proliferation and induce apoptosis in ovarian cancer cell lines [11,12,27]. However, there is little convincing evidence for an association between 25(OH)D and the risk of developing ovarian cancer in a pooled analysis [28] and a meta-analysis [29]. Furthermore, the mechanism on antitumor of vitamin D on ovarian cancer is unclearly understood. Some experimental studies strongly suggest that 1α,25(OH)_2_D_3_ arrested ovarian cancer cells in G1 and G2/M phase by modulating GADD45 and p27 [6,30]. Our previous study indicated that 1α,25(OH)_2_D_3_ enhances the therapeutic effects of carboplatin by altering the cell cycle and increasing apoptosis through changes in reactive oxygen species and mitochondria membrane potential in SKOV-3 cells [12]. These findings demonstrated that vitamin D inhibited proliferation of ovarian cancer cells. The findings firstly presented in this study indicated that 1α,25(OH)_2_D_3_ decreased cell migration through inhibiting TGF-β1-induced EMT in human ovarian cancer SKOV-3 cells. It was in colon cancer cells that 1α,25(OH)_2_D_3_ inhibited TGF-β1/β2-increased invasion and migration by inhibiting the switch of cadherin and expression of EMT-related transcription factors. 1α,25(OH)_2_D_3_ also inhibited the secretion of MMP-2 and MMP-9 and increased expression of F-actin induced by TGF-β1/β2 in colon cancer cells [15]. 1α,25(OH)_2_D_3_ and its analogs inhibit the migration and invasion of tumor cells by regulating changes in the cell–extracellular matrix interaction as well as by promoting cell–cell contact in breast, prostate and colorectal cancer cells [31,32]. Taken together, the restraint of EMT might be one of the mechanisms underlying the anti-metastasis effect of 1α,25(OH)_2_D_3_ in cancer cells.

Transcriptional factor β-catenin aggregating toward the nucleus is also considered as a sign of EMT [33]. In addition, E-cadherin could mediate the migration of β-catenin from nucleus to cytoplasm [34]. In this study, both β-catenin gathered to the nucleus and E-cadherin decreased in the cytoplasm were observed in SKOV-3 cells induced by TGF-β1. At the same time, the expression of Slug and Snail were increased in nuclei of ovarian cancer cells. Moreover, 1α,25(OH)_2_D_3_ significantly reversed the expression of EMT-related transcription factors induced by TGF-β1. Chen et al. also reported that increased TGF-β1/β2 expression of EMT-related transcription factors in colon cancer cells was also inhibited by 1α,25(OH)_2_D_3_ [15]. In addition, Snail repressed expression of VDR, resulting in reduction of the anticancer effects of 1α,25(OH)_2_D_3_ [35]. In another study for colon cancer, the author reported that expression of VDR was negatively correlated with those of snail and ZEB1 in the cancer tissue [36]. We found that 1α,25(OH)_2_D_3_ could change the localization of Slug, Snail, and β-cateinin and inhibited their expression in SKOV-3 cells exposed to TGF-β1, which was associated with the increase of VDR. The data suggested that vitamin D treatment strategies play their protective roles in ovarian tumor progression, by increasing VDR expression.

## 4. Experimental Section

### 4.1. Cell Culture and Treatment

Human ovarian epithelial adenocarcinoma cell lines SKOV-3 were obtained from the Type Culture Collection of the Chinese Academy of Sciences (Shanghai, China), and maintained in Roswell Park Memorial Institute (RPMI) 1640 (Invitrogen Carlsbad, San Diego, CA, USA) with 10% fetal bovine serum (FBS, Sigma-Aldrich Chemie GmbHFBS, Steinheim, Germany), 100 U/mL penicillin, and 100 µg/mL streptomycin (Beyotime Biotechnology, Shanghai, China) in a humidified atmosphere of 5% CO_2_ at 37 °C.

The cells were cultured at approximately 80% confluency and starved in serum-free RPMI 1640 overnight. After being removed from culture medium, SKOV-3 cells were treated with different factors, respectively. The cells in control group were treated with vehicle (0.1% ethanol), TGF-β1 group (10 ng/mL, PEPROTECH, Princeton, NJ, USA), VD group treated with different concentrations of 1α,25(OH)_2_D_3_ (1, 10, 100 nmol/L) , which was purchased from Sigma (Sigma-Aldrich Chemie GmbH, Steinheim, Germany) and the cells in TGF-β1 + VD group were adminstrated with combination TGF-β1 (10 ng/mL) and 1α,25(OH)_2_D_3_ (100 nmol/L).

### 4.2. Wound Healing Assay

The migration capacities of SKOV-3 cells were assessed by wound healing assay. Cells were plated, and serum-starved for 24 h after the cells adherent on culture dish about 12 h. A wound was created by scraping the cells with a sterile 1000 μL pipette tip in the middle of the culture well. Then, the dish was softly washed in phosphate-buffered saline (PBS) and put in a culture medium with different factors. The wound closure photographs were captured using a microscope (magnification of ×40, CKX41F, Olympus, Tokyo, Japan) equipped with a digital camera. A measure was taken of it, and then the average value was calculated. Data are presented as migration index (%) = [(the initialized width of the scratch) − (the final width of the scratch)]/(the initialized width of the scratch). Data points in figures represent three independent experiments.

### 4.3. Live Cell Imaging System

SKOV-3 cells during logarithmic phase were planted in 24-well plates (Thermo Fisher Scientific, Waltham, MA, USA) for 12 h, and removed from culture medium. Then, cells were starved in serum-free RPMI 1640 for 24 h and treated with vehicle, 100 nmol/L of 1α,25(OH)_2_D_3_, 10 ng/mL of TGF-β1, or combination TGF-β1 with 1α,25(OH)_2_D_3_, respectively. SKOV-3 cells were incubated in the Live Cell Imaging System (Cell^R, Olympus, Tokyo, Japan) to monitor their growth in real time for 72 h.

### 4.4. Invasion Assay

SKOV-3 cells were seeded in the the 24-well BD Biocoat Matrigel Invasion Chambers (BD Biosciences, Franklin Lakes, NJ, USA) with a vehicle, indicated concentration of TGF-β1 or 1α,25(OH)_2_D_3_ or combination of them, respectivedly. The lower chamber was supplemented with RPMI1640 culture containing 10% FBS. After the cells were cultured for 24 h, non-invading cells were carefully removed with a cotton swab. The cells on the bottom of inserts were fixed with 70% ethanol and were stained with Crystal Violet (c0121, Beyotime Biotechnology, Shanghai, China) for 3 min. The number of cells penetrating the membrane were calculated and pictures were taken under the microscope (×40, CKX41F, Olympus, Tokyo, Japan). Data points in figures represent three independent experiments.

### 4.5. Immunofluoresecence Staining

SKOV-3 cells were grown on Cover slides (Thermo Fisher Scientific, Waltham, MA, USA), which were put into 24-well plates. After the cells adhered on the plate, cells were treated with a vehicle, indicating concentration of TGF-β1 or 1α,25(OH)_2_D_3_, or combination of them, respectively. After 24 h, cells were washed with cool PBS twice, and fixed in 4% paraformaldehyde for 20 min at 4 °C. Then, cells were permeabilized with 0.1% Triton at 4 °C for 15 min, and nonspecific binding was blocked with 1% FBS in confining liquid for 1 h at room temperature (RT). Next, the cells were incubated with primary antibodies: E-cadherin (32A8) Mouse mAb (1:100, #5296, Cell Signaling Technology, Irvine, CA, USA), Vimentin (D21H3) XP^®^ Rabbit mAb (1:100, #5741, Cell Signaling Technology), β-catenin (6B3) Rabbit mAb (1:100, #9582, Cell Signaling Technology), Slug (C19G7) Rabbit mAb (1:100, #9585, Cell Signaling Technology), Snail (C15D3) Rabbit mAb (1:50, #3879, Cell Signaling Technology), mAb Anti-Vitamin D Receptor Antibody (1:100, NBP1-51322, Novus Biologicals, Littleton, CO, USA). After being incubated with primary antibodies at 4 °C overnight, the cells were stained with secondary antibody IGg-Cy5 (1:1000, #4412, Cell Signaling Technology) in the dark room for 1.5 h and washed with PBS for 3 min (3 times) in a horizontal earthquake shaking bed. Nuclei were labeled with DAPI for 20 min. Images were captured with Confocal Laser Scanning Microscope (TCS SP2, Leica, Wetxlar, Germany).

### 4.6. Western Blot

After being treated with a vehicle, indicating concentration of TGF-β1 or 1α,25(OH)_2_D_3_, or a combination of them, respectively, SKOV-3 cells were harvested and lysed for total cellular protein extraction with RIPA buffer (p0013, Beyotime Biotechnology, Shanghai, China). The cells were centrifuged at 12,000 rpm for 30 min and the supernatants were collected. The protein concentration of lysate was quantified using a Bicinchonininc acid (BCA) protein assay kit (P0012, Beyotime Biotechnology, Shanghai, China). Equal amounts of total proteins (30 µg) were loaded onto 10% sodium dodecyl sulphate—polyacrylamide gel (SDS-PAGE) (P0012A, Beyotime Biotechnology) and the proteins were electrophoretically transferred onto a polyvinylidene fluoride (PVDF) membrane (Millipore, Boston, MA, USA). After being blocked with 5% skimmed milk, the membranes were incubated with primary antibodies of E-cadherin (1:700), Vimentin (1:800), Snail (1:700), Slug (diluted 1:700), β-catenin (1:100) or VDR (1:100) at 4 °C for overnight, respectively. Then, cells were washed 3 times by Phosphate Buffered Saline with Tween-20 (PBST) and incubated with anti-mouse or anti-rabbit horseradish peroxidase-conjugated secondary antibodies at RT for 1 h, and then washed 3 times by PBST again. The detection of the antigen–antibody complex was visualized using chemiluminescence (Immobilon ECL) reagent (Millipore). The indicated protein was quantified with gray value to identify the respective expression of targeted protein relative to β-actin (as the loading control). Data points in figures represent three independent experiments.

### 4.7. Statistical Analysis

Statistical analysis was performed using SPSS, version 17.0 for Windows (SPSS, Inc., Chicago, IL, USA). The data of the experiments were presented as means and standard deviation and analyzed with Student’s *t*-test and ANOVA.

## 5. Conclusions

1α,25(OH)_2_D_3_ not only inhibited the invasion and the migration of SKOV-3 cells, but also promoted the acquisition of an epithelial phenotype of SKOV-3 cells treated with TGF-β1. We discovered that 1α,25(OH)_2_D_3_ increased the expression of epithelial marker E-cadherin while decreasing the level of mesenchymal marker, Vimentin, which was associated with the elevated expression of VDR. Moreover, 1α,25(OH)_2_D_3_ reduced the expression level of EMT-related transcription factors, such as slug, snail and β-catenin. These results indicate that 1α,25(OH)_2_D_3_ suppresses the metastasis of ovarian cancer cells by regulating EMT, implying that 1α,25(OH)_2_D_3_ might be a potential therapeutic agent for the treatment of ovarian cancer.

## Supplementary Materials

Supplementary materials can be found at www.mdpi.com/1422-0067/17/8/1285/s1.

## Abbreviations

EMT	Epithelial-mesenchymal transition
1α,25(OH)_2_D_3_	1α,25-dihydroxyvitamin D
VDR	vitamin D receptor
25(OH)D	25-dihydroxyvitamin D
