# Supplementary material for: 1α,25(OH)2D3 Suppresses the Migration of Ovarian Cancer SKOV-3 Cells through the Inhibition of Epithelial–Mesenchymal Transition
Source: Int J Mol Sci. 2016 Aug 19;17(8):1285. doi: 10.3390/ijms17081285 (PMC5000682; doi:10.3390/ijms17081285)
Supplement: Supplementary file 1 [file ijms-17-01285-s001.pdf]

Supplementary Material: 1α,25(OH)$_2$D$_3$ Suppresses the Migration of Ovarian Cancer SKOV-3 Cells through the Inhibition of Epithelial–Mesenchymal Transition

Yong-Feng Hou, Si-Hai Gao, Ping Wang, He-Mei Zhang, Li-Zhi Liu, Meng-Xuan Ye, Guang-Ming Zhou, Zeng-Li Zhang and Bing-Yan Li

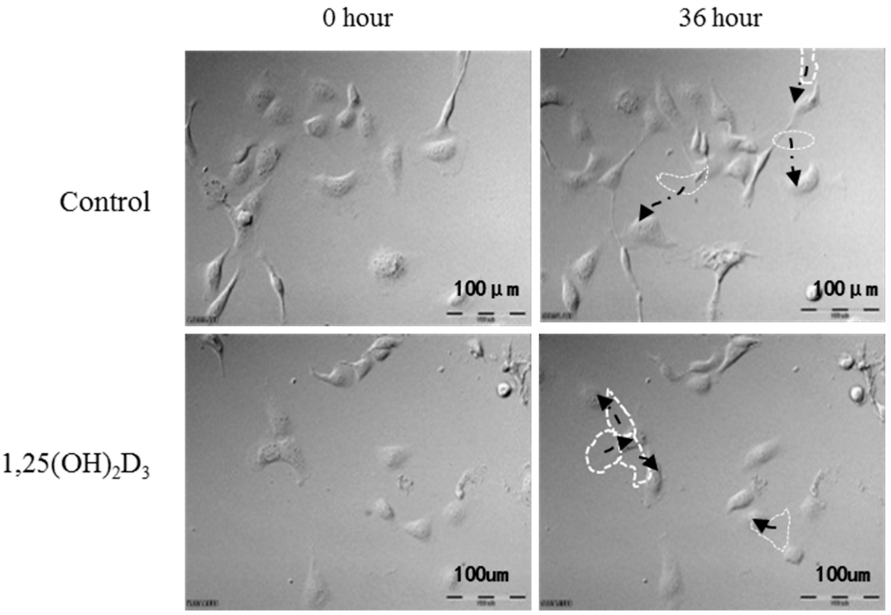

Figure S1. 1α,25(OH)$_2$D$_3$ slowed down motion tracking of SKOV-3 cells compared with control (Arrows refer to the movement track of SKOV-3 cells).

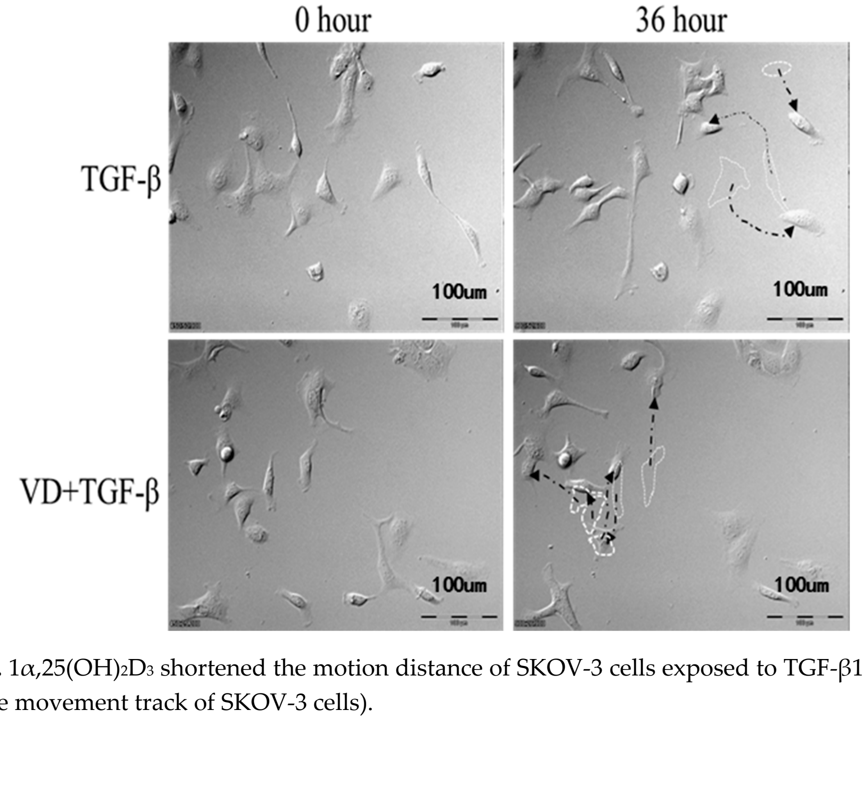

Figure S2. 1α,25(OH)$_2$D$_3$ shortened the motion distance of SKOV-3 cells exposed to TGF-β1 (Arrows refer to the movement track of SKOV-3 cells).
